# Point‐of‐care gastrointestinal ultrasound in inflammatory bowel disease: An accurate alternative for disease monitoring

**DOI:** 10.1002/jgh3.12269

**Published:** 2019-10-09

**Authors:** Dharshan Sathananthan, Arvind Rajagopalan, Lucinda Van De Ven, Serena Martin, James Fon, Samuel Costello, Robert V Bryant

**Affiliations:** ^1^ Gastroenterology Department Queen Elizabeth Hospital Adelaide South Australia Australia; ^2^ School of Medicine The University of Adelaide Adelaide South Australia Australia

**Keywords:** gastrointestinal ultrasound, ileocolonoscopy, inflammatory bowel disease, point‐of‐care ultrasound

## Abstract

**Background and Aim:**

Point‐of‐care ultrasound (POCUS) is a noninvasive alternative to ileocolonoscopy for monitoring disease activity in inflammatory bowel disease (IBD) but is underutilized in practice. Accuracy data are needed to engender clinician confidence in POCUS and increase uptake. The aim of this study was to evaluate the accuracy of POCUS compared to ileocolonoscopy in detecting active disease and extent in patients with IBD.

**Methods:**

A prospective, blinded study was performed at a single tertiary center in South Australia between May 2017 and May 2018. Consecutive patients with a formal diagnosis of IBD who underwent both POCUS and ileocolonoscopy within 30 days of one another, performed to evaluate IBD disease activity, were eligible for participation. The accuracy of POCUS compared to ileocolonoscopy was assessed using sensitivity, specificity, and Cohen's kappa coefficient analyses.

**Results:**

A total of 74 patients were included in the final analysis, 35 (47%) of whom had Crohn's disease and 39 (53%) ulcerative colitis; 37 subjects (50%) underwent a POCUS and ileocolonoscopy on the same day. POCUS demonstrated 91% sensitivity and 83% specificity for detecting endoscopically active IBD, correlating with a positive predictive value (PPV) of 89%, a negative predictive value (NPV) of 86%, and a kappa coefficient of 0.74 (88%). POCUS defined disease extent with 87% sensitivity and 81% specificity, correlating with a PPV of 85% and NPV of 83% and a kappa coefficient of 0.70 (85%).

**Conclusion:**

POCUS is accurate in defining disease activity and extent in IBD compared to ileocolonoscopy. POCUS represents an appealing, noninvasive alternative to ileocolonoscopy for monitoring disease activity in IBD.

## Graphical abstract

Point of care ultrasound is incorporated into routine IBD assessment in many European centers, however uptake in Australia has been slow. This article evaluates the accuracy of point of care ultrasound in monitoring disease activity in a local cohort of IBD patients. Point of care ultrasound was found to be accurate for monitoring disease activity and extent in IBD compared to the gold standard, ileocolonoscopy.

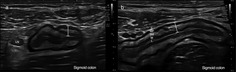

## Introduction

Expansion in the armamentarium of effective treatment options for inflammatory bowel disease (IBD) has evolved goals of therapy.^1^ Beyond symptom resolution, mucosal healing is now aggressively pursued as a therapeutic target in IBD as it is associated with decreased rates of hospitalization, surgery, steroid use, and risk of malignancy.^1^, ^2^, ^3^, ^4^, ^5^, ^6^

Clinical symptoms correlate poorly with inflammatory burden in IBD.^7^ Consequently, objective assessment of disease activity is recommended to guide ongoing management.^1^ The gold standard for assessing disease activity in IBD is ileocolonoscopy.^1^ However, ileocolonoscopy is invasive and is associated with risks to patients, not to mention the discomfort and inconvenience of bowel preparation.^8^ Furthermore, the frequency at which ileocolonoscopy is recommended for monitoring IBD poses an enormous economic burden on health systems. Alternatives to ileocolonoscopy are therefore necessary to objectively monitor IBD disease activity and inform management decisions.

Point‐of‐care ultrasound (POCUS) is an ideal modality to assess disease activity in IBD as it is accurate, accessible, noninvasive, and acceptable to patients.^9^ POCUS has been shown to have comparable accuracy in terms of establishing disease activity and extent for both ulcerative colitis (UC) and Crohn's disease (CD) compared to both ileocolonoscopy and other imaging modalities, including computed tomography (CT) and magnetic resonance imaging (MRI).^1^, ^9^, ^10^, ^11^ POCUS has also been shown to closely correlate with endoscopic mucosal healing in both UC and CD.^12^, ^13^, ^14^ Moreover, POCUS is useful in identification of complications such as abscesses and fistulae.^9^ A unique feature of POCUS is its ability to be performed at the bedside to facilitate real‐time clinical decision‐making to triage and expedite appropriate further investigations and management.^14^

In many European centers, POCUS is incorporated into routine IBD assessment.^15^ However, in other countries, such as Australia, uptake of POCUS has been slow, limited by a lack of awareness, an absence of training opportunity, a perception of operator dependence, and uncertainty about how to incorporate POCUS into established algorithms of IBD management.^7^, ^16^ The primary objective of this study was therefore to assess the accuracy of POCUS as a noninvasive modality of predicting disease activity in patients with IBD compared to the gold standard, ileocolonoscopy. The secondary objectives of this study were to assess the accuracy of POCUS in defining disease extent compared to ileocolonoscopy and, finally, to compare the accuracy of POCUS to the biomarker fecal calprotectin (FC) in predicting disease activity.

## Methods

### *Subjects*

Consecutive patients with a formal diagnosis of IBD managed by a tertiary IBD service who underwent POCUS during routine clinical care were invited to participate in this prospective study between May 2017 and May 2018. POCUS was requested by the treating clinician to evaluate the activity and extent of IBD in order to inform treatment decisions. POCUS examination findings were compared to the findings of ileocolonoscopy, concurrently requested by the treating clinician. All patients included in the study underwent both POCUS and ileocolonoscopy within 30 days of one another.

Participants were provided with a written information sheet prior to provision of informed consent. All patients undergoing POCUS examination with a formal diagnosis of IBD who provided consent were eligible for inclusion in this study. Patients with isolated rectal disease were included in this study despite the known limitations of POCUS to obtain adequate views of the rectum. Patients who did not have a comparative ileocolonoscopy performed within 30 days of a POCUS or those who had a significant change in clinical condition or therapy in the interval between POCUS and ileocolonoscopy were excluded from this study.

### *POCUS examination*

POCUS was performed by a single, accredited sonographer (as recognized by the Australian Gastrointestinal Network of Intestinal Ultrasound) with 2 years of experience, including >600 dedicated gastrointestinal ultrasound scans. POCUS was performed in a variety of clinical settings, including the endoscopic suite, hospital ward, and outpatient clinic. The sonographer was blinded to the results of the comparative ileocolonoscopic examination.

**Figure 1:**
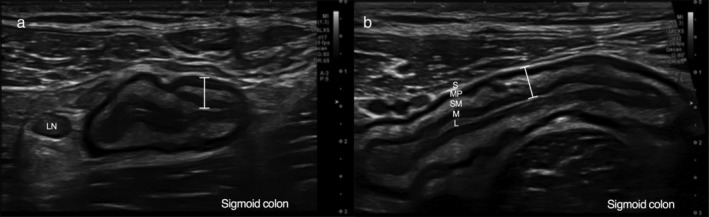
Colonic inflammation detected using point‐of‐care gastrointestinal ultrasound. (a) Transverse section of inflamed sigmoid colon in a patient with Crohn's colitis characterized by increased wall thickness (5.5 mm, measured using marker), abnormal wall stratification with submucosal prominence, and an enlarged lymph node within mesenteric hyperechogenicity reflective of fibrofatty proliferation. Note that Doppler signal is not shown. (b) Longitudinal section of same inflamed sigmoid colon in a patient with Crohn's colitis with a marker again showing increased wall thickness (5.5 mm). The bowel wall layers are annotated: L, luminal interface, white; M, mucosa, black; SM, submucosa, white; MP, muscularis propria, black; S, serosa, white. Note that Doppler signal is not shown.

POCUS was performed using a Toshiba Aplio 500 ultrasound unit with a low‐frequency (1–6 MHz) and high‐frequency (3–11 MHz) transducer. No specific patient preparation was used. A transabdominal approach was used for all assessments, with systematic evaluation of the gastrointestinal tract.^17^ Each assessment lasted 10–20 min, and the results of the examination were recorded on a standardized reporting template. In the absence of a validated gastrointestinal ultrasound scoring system for IBD disease activity, a recently proposed scoring system by Medellin *et al*. was prospectively applied to POCUS studies by the sonographer.^18^, ^19^ Active disease for both CD and UC was defined as an increase in bowel wall thickness >3 mm accompanied by an increase in Doppler color signal in the bowel wall (Fig. 1).^18^ In the setting of active disease, sonographic disease extent was also recorded according to the Montreal criteria (E1‐3 for UC and L1‐3 for CD).^20^

### *Ileocolonoscopic examination*

Ileocolonoscopy was performed by two experienced IBD‐focused gastroenterologists (Samuel Costello, James Fon). Each of the proceduralists was blinded to the results of the POCUS examination. Ileocolonoscopy was performed under sedation following a standard bowel preparation. On completion of ileocolonoscopy, the endoscopic Mayo score for patients with UC or simple endoscopic score for CD (SES‐CD) was prospectively recorded for each patient by the endoscopist. Active endoscopic disease activity for CD was defined as the presence of ulceration in any bowel segment.^1^ For UC, active endoscopic disease was defined as a Mayo score of ≥1.^1^ A subanalysis for UC was also conducted where active disease was defined as a Mayo score > 1. Endoscopic disease extent was also recorded according to Montreal criteria.^20^

### *Data collection*

Data were collected on patient demographics, clinical information including disease phenotype, Montreal classification, and current treatment regimen. FC levels were also recorded when prospectively requested by the treating clinician and performed within 30 days of ileocolonoscopy. An FC level of >50 μg/g was used to define active disease.^21^

### *Ethics*

The study was approved by the Central Adelaide Local health Network Research Ethics Committee (R20170209, February 2017).

## Statistical analysis

The accuracy of POCUS and FC to detect active disease and to define disease compared to ileocolonoscopy was determined by calculating the sensitivity, specificity, positive predictive value (PPV), and negative predictive value (NPV) within a 95% confidence interval. Agreement between comparative diagnostic modalities was assessed using kappa's coefficient. All analyses were conducted using Stata (StataCorp. 2015. Stata Statistical Software: Release 14; StataCorp LP, College Station, TX, USA).

## Results

**Figure 2:**
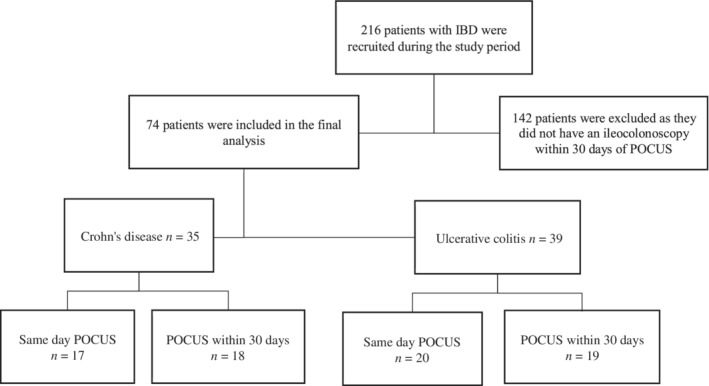
Study flow chart. IBD, inflammatory bowel disease; POCUS, point‐of‐care ultrasound.

A total of 216 patients were recruited to participate in this study, 142 of whom were excluded as they did not undergo ileocolonoscopy within 30 days of POCUS examination (Fig. 2). A total of 74 patients were included in the final analysis; 39 (53%) were male, 35 (47%) had CD, and the median age was 45 years (interquartile range [IQR] 33–57) (Table 1). Endoscopically active disease was evident in 19 (54%) of the 35 patients with CD and 24 (65%) of the 39 patients with UC. The median interval between POCUS and ileocolonoscopy was 0 days (IQR 0–10).

**Table 1:** Clinical and demographic characteristics of study cohort

	Overall	CD	UC
Number of patients (*n*, %)	74	35 (47)	39 (53)
Age (mean ± SD/median, IQR)	47 ± 16/45 (33, 57)	46 ± 13/43 (33, 54)	47 ± 18/42 (31, 57)
Male (*n*, %)	39 (52)	15 (43)	24 (62)
Disease duration—years (median, IQR)	6 (2, 16)	9 (1, 21)	6 (2, 11)
Montreal classification^20^ (*n*, %)	A1 6 (8)	A1 3 (9), L1 8 (23), B1 18 (51)	A1 3 (8), E1 3 (8)
	A2 38 (51)	A2 18 (51), L2 15 (43), B2 5 (14)	A2 20 (74), E2 24 (62)
	A3 28 (37)	A3 13 (37), L3 12 (34), B3 10 (29)	A3 15 (38), E3 8 (20)
		P6 (17)	
Disease activity		SES CD 0, 46 (%)	Mayo 0, 38 (%)
		SES CD ≥ 1, 54 (%)	Mayo 1, 16 (%)
		SES CD mean 6 ± 7, median 4, IQR (1, 6)	Mayo 2, 28 (%)
			Mayo 3, 18 (%)
			Mean 1.3 ± 1.16, median 1
Medications			
5‐Aminosalicyclate (*n*, %)	31 (42)	5 (14)	26 (67)
Immunomodulator (*n*, %)	30 (41)	16 (46)	14 (36)
Biologic (*n*, %)	23 (31)	13 (37)	10 (26)
Corticosteroids (*n*, %)	9 (12)	5 (14)	4 (10)
No treatment (*n*, %)	5 (7)	4 (11)	1 (2)

Complete case analysis presented. Percentages calculated based on the total patient cohort and where data are unavailable may not equal 100%.

CD, Crohn's disease; IQR, interquartile range; Mayo, Mayo endoscopic subscore; SES CD, simple endoscopic score for Crohn's disease; UC, ulcerative colitis.

### *Accuracy of POCUS for IBD disease activity assessment*

#### *POCUS within 30 days of ileocolonoscopy*

For UC, POCUS demonstrated 92% sensitivity and 86% specificity in detecting endoscopically active disease (defined as an endoscopic Mayo subscore of ≥1), correlating with a PPV of 92%, NPV of 86%, and a kappa coefficient of 0.77 (89%) (Table 2). Similar accuracy was demonstrated when active UC was defined as an endoscopic Mayo subscore of >1. There were three patients with isolated ulcerative proctitis included in the study, each of whom had active disease at ileocolonoscopy that was accurately identified by POCUS. For CD, POCUS demonstrated 90% sensitivity and 81% specificity in detecting endoscopically active disease, correlating with a PPV of 85%, an NPV 87%, and a kappa correlation of 0.71 (86%). Overall, POCUS demonstrated 91% sensitivity and 83% specificity for detecting endoscopically active IBD, correlating with a PPV of 89%, an NPV of 86%, and a kappa coefficient of 0.74 (88%) (Table 2).

**Table 2:** Accuracy of POCUS in detecting active disease compared to ileocolonoscopy

Ileocolonoscopy	POCUS									
	Sensitivity (%)		Specificity (%)		PPV (%)		NPV (%)		Kappa coefficient	
	<30 days	Same‐day	<30 days	Same‐day	<30 days	Same‐day	<30 days	Same‐day	<30 days	Same‐day
UC (Mayo ≥ 1)	92 (73–99)	100 (77–100)	86 (57–98)	100 (54–100)	92 (73–99)	100 (77–100)	86 (57–98)	100 (54–100)	0.77 (89%)	1 (100%)
UC (Mayo > 1)	94 (72–100)	100 (66–100)	85 (57–98)	100 (54–100)	89 (67–99)	100 (66–100)	92 (64–100)	100 (54–100)	0.80 (90%)	1 (100%)
CD	90 (67–99)	91 (59–100)	81 (54–96)	83 (35–100)	85 (62–97)	91 (59–100)	87 (60–98)	83 (36–100)	0.71 (86%)	0.74 (88%)
Overall	91 (78–97)	96 (80–100)	83 (65–94)	92 (62–100)	89 (75–96)	96 (80–100)	86 (68–96)	92 (62–100)	0.74 (88%)	0.87 (95%)

CD, Crohn's disease; NPV, negative predictive value; POCUS, point‐of‐care gastrointestinal ultrasound; PPV, positive predictive value; UC, ulcerative colitis.

#### *POCUS on same day as ileocolonoscopy*

Of the 74 patients included in this study, 37 (50%) underwent blinded POCUS and ileocolonoscopy on the same day. For UC, POCUS was found to be 100% accurate in detecting active endoscopic disease, defined as either endoscopic Mayo subscore of ≥1 or >1 (sensitivity 100%, specificity 100%, PPV 100%, NPV 100%, kappa coefficient 1 [100%]).

For CD, same‐day POCUS demonstrated 91% sensitivity and 83% specificity for endoscopically active disease, correlating with a PPV or 91%, an NPV of 83%, and a kappa coefficient of 0.74 (88%). Overall, same‐day POCUS demonstrated 96% sensitivity and 92% specificity for endoscopically active disease, correlating with a PPV of 96%, an NPV 92%, and a kappa coefficient of 0.87 (95%) (Table 2).

### *Accuracy of POCUS for IBD disease extent*

Disease extent using the Montreal criteria was defined only for those with active disease on ileocolonoscopy in the study. Of the patients, 24 (61%) with UC had endoscopically active disease (Mayo subscore ≥1), of whom POCUS correctly defined disease extent in 22 (91%). For UC, POCUS demonstrated 92% sensitivity and 80% specificity in defining disease extent, correlating with a PPV of 88%, an NPV 86%, and a kappa coefficient of 0.7 (87%). Of the 19 (54%) patients with CD with endoscopically active disease, data on disease extent were available for 16 patients. POCUS correctly defined disease extent in 13 of 16 (81%) patients with CD. For CD, POCUS demonstrated 81% sensitivity and 81% specificity in defining disease extent, correlating with a PPV of 81%, NPV of 81%, and a kappa coefficient of 0.6 (81%). Overall, the sensitivity, specificity, PPV, and NPV of POCUS in defining disease extent in IBD were 87, 81, 85, and 83%, respectively. The correlation compared to ileocolonoscopy was good, with a kappa coefficient of 0.7 (85%) (Table 3).

**Table 3:** Accuracy of POCUS in defining inflammatory bowel disease extent compared to ileocolonoscopy

		Sensitivity (%)	Specificity (%)	PPV (%)	NPV (%)	Kappa coefficient (%)
POCUS	UC (*n* = 24)	92 (73–99)	80 (52–96)	83 (73–95)	86 (73–96)	0.7 (87)
	CD (*n* = 16)	81 (54–96)	81 (54–96)	81 (60–93)	81 (60–93)	0.6 (81)
	Overall	87 (73–96)	81 (63–93)	85 (74–92)	83 (68–92)	0.7 (85)

Only patients with active disease (Mayo endoscopic subscore ≥1 and simplified endoscopic score for Crohn's disease ≥1 and ulceration) were included in assessment of disease extent.

CD, Crohn's disease; NPV, negative predictive value; POCUS, point‐of‐care gastrointestinal ultrasound; PPV, positive predictive value; UC, ulcerative colitis.

### *Comparative accuracy of FC for IBD disease activity*

Thirty patients (40%, 18 UC, 12 CD) underwent FC testing within 30 days of ileocolonoscopy. For UC, an FC cut‐off of >50 μg/g detected only four (44%) of nine patients with endoscopically active disease (Mayo ≥ 1). Accordingly, FC demonstrated a sensitivity of 44% and a specificity of 100% for endoscopically active UC, correlating with a PPV of 100%, an NPV of 64%, and a kappa coefficient of 0.4 (72%). For CD, an FC cut‐off of >50 μg/g detected every patient (6/6) with endoscopically active disease. Accordingly, FC demonstrated a sensitivity of 100% and a specificity of 17% for endoscopically active CD, correlating with a PPV of 55%, an NPV of 100%, and a kappa coefficient of 0.2 (58%). Although definitive conclusions are limited by power, FC was found to be less accurate than POCUS for IBD disease activity assessment in the studied cohort.

## Discussion

POCUS was found to be accurate compared to ileocolonoscopy in assessing disease activity and extent in the routine monitoring of patients with IBD. POCUS was more accurate for defining disease activity and extent in patients with UC and was more accurate than FC in predicting disease activity. The study findings inform clinicians of the utility of POCUS as a noninvasive modality for disease assessment in IBD that should be incorporated into the routine care of patients with IBD in the “treat to target” era.

The accuracy of POCUS is well established in Europe where it is routinely incorporated into IBD care.^9^ Moreover, there are appreciable advantages of POCUS that make it a favored monitoring tool in IBD. It is noninvasive, safe, tolerable, does not require prior preparation, and can be performed in real time, allowing treatment decisions to be made within the same clinical encounter. However, uptake of the technique in North America and Australasia has been slow, hampered by lack of awareness, training opportunities, and a broad perception that ultrasound is operator dependent.^9^, ^16^ Imaging preference is also driven by reimbursement, with cross‐sectional imaging such as computed tomography and MRI fetching higher remuneration. Furthermore, there has been a paucity of local data informing clinicians of the accuracy and utility of POCUS in IBD care.

This study provides sound evidence that POCUS is accurate in assessing both IBD disease activity and extent compared to ileocolonoscopy in an Australian cohort. The accuracy data are comparable to those of previous studies, mostly conducted in Europe, which have shown that POCUS has an overall sensitivity of 85% and specificity of 91% in detecting active disease, compared to ileocolonoscopy in patients with CD, and a sensitivity of 74% and specificity of 93% in patients with UC (proximal to the rectum).^9^ Interestingly, in this study, POCUS was found to be more accurate for predicting active disease and extent in UC than in CD. A small number of patients with isolated proctitis were included in this study to mirror “real‐world” practice, and POCUS was able to accurately identify active disease. There are a few papers reporting the utility of POCUS in the assessment of UC.^8^, ^9^, ^10^, ^13^ The paucity of data relating to the utility of POCUS in the assessment of UC may be because POCUS is generally not recommended as a tool for assessing isolated rectal disease due to unreliable attainment of adequate views into the pelvis via a transabdominal approach.^9^ Moreover, the perceived ease and safety of performing a flexible sigmoidoscopy to assess disease activity limits the clinical consideration of alternative approaches. This perception, however, may be inaccurate as a flexible sigmoidoscopy is not risk free, requires preparation, is typically performed with sedation, and may not be accepted by all patients. In a nationwide French study assessing the acceptability of monitoring tools in IBD, flexible sigmoidoscopy was considered the least acceptable monitoring tool by patients with UC compared to venipuncture, FC, and ileocolonoscopy.^22^ Our findings suggest that POCUS can be effectively used as an alternative tool to monitor disease activity in patients with UC with disease extending beyond the rectum.

FC is a noninvasive biomarker, which is well established in IBD management algorithms. FC has been shown to be responsive to changes in disease activity following adjustment in therapy, with a good NPV for active disease.^21^, ^23^, ^24^, ^25^ There are some limitations to FC, however, as levels of FC vary on a daily basis, the cut‐offs used to define active disease have varied in the literature, and FC may not in fact correlate with mucosal healing.^21^ This is particularly true for mild elevations of FC of 50–150 μg/g.^21^ In this study, POCUS was found to be more accurate that FC in predicting endoscopic disease activity in patients with UC. Surprisingly, an FC cut‐off of 50 μg/g was relatively insensitive in detecting active disease in the UC cohort. Not only was POCUS more accurate than FC at detecting disease activity, but it also provided additional information on disease extent. Small patient numbers limit the capacity to draw firm conclusions from these data. Further studies are warranted to compare the accuracy of FC and POCUS in assessing IBD disease activity (using different FC cut‐offs) and to assess whether gains are made by using both tests as a composite noninvasive approach.

Strengths of this study include prospective methodology coupled with blinded performance of POCUS and ileocolonoscopy. Half of the patients underwent POCUS and ileocolonoscopy on the same day, which adds credence to the evaluation of comparative accuracy. To our knowledge, this is the first study to compare the accuracy of POCUS to FC in detecting disease activity. Weaknesses of this study include relatively small patient numbers comprised of a heterogenous population, which limited the power and ability to draw firm conclusions from subanalyses. The inherent issue with comparing different modalities of disease activity assessment in IBD must be acknowledged. Each modality incorporates distinct measures that may not be comparable and are merely surrogate metrics for inflammation. For example, endoscopy evaluates only mucosal inflammation using features of erythema and ulceration without direct assessment of blood flow. On the other hand, gastrointestinal ultrasound evaluates transmural inflammation without readily evaluating mucosal ulceration. The study was conducted in a single center with a single operator, which may limit the generalizability of findings. Utility of an unvalidated scoring system incorporating both wall thickness and Doppler signal, without evaluation of other features of inflammation such as mesenteric hyperechogenicity and wall stratification, is a weakness of this study, yet there does not exist a validated metric for sonographic disease activity in IBD.^9^ Finally, disease extent was assessed using the Montreal criteria, and further information may have been obtained if extent was defined by disease segment with correlating segmental accuracy analyses.

In summary, POCUS is a noninvasive tool for monitoring IBD disease activity, which is accurate compared to ileocolonoscopy. POCUS may be performed at the bedside to facilitate real‐time clinical decision‐making and warrants reappraisal for use in routine IBD management outside of Europe.
